# Different dynamical behaviors induced by slow excitatory feedback for type II and III excitabilities

**DOI:** 10.1038/s41598-020-60627-w

**Published:** 2020-02-27

**Authors:** Zhiguo Zhao, Li Li, Huaguang Gu

**Affiliations:** 1 School of Science, Henan Institute of Technology, Xinxiang, 453003 China; 2 Guangdong Key Laboratory of Modern Control Technology, Guangdong Institute of Intelligent Manufacturing, Guangzhou, 510070 China; 3 School of Aerospace Engineering and Applied Mechanics, Tongji University, Shanghai, 200092 China

**Keywords:** Nonlinear phenomena, Dynamical systems, Dynamical systems

## Abstract

Neuronal excitability is classified as type I, II, or III, according to the responses of electronic activities, which play different roles. In the present paper, the effect of an excitatory autapse on type III excitability is investigated and compared to type II excitability in the Morris-Lecar model, based on Hopf bifurcation and characteristics of the nullcline. The autaptic current of a fast-decay autapse produces periodic stimulations, and that of a slow-decay autapse highly resembles sustained stimulations. Thus, both fast- and slow-decay autapses can induce a resting state for type II excitability that changes to repetitive firing. However, for type III excitability, a fast-decay autapse can induce a resting state to change to repetitive firing, while a slow-decay autapse can induce a resting state to change to a resting state following a transient spike instead of repetitive spiking, which shows the abnormal phenomenon that a stronger excitatory effect of a slow-decay autapse just induces weaker responses. Our results uncover a novel paradoxical phenomenon of the excitatory effect, and we present potential functions of fast- and slow-decay autapses that are helpful for the alteration and maintenance of type III excitability in the real nervous system related to neuropathic pain or sound localization.

## Introduction

Action potentials related to ionic currents play important roles in neural information transmission and coding^1–4^. Neurons have different expressions of ion channels, and can produce action potentials via different responses to stimulations^1^. Based on the responses of the resting state to constant depolarization current stimulations, excitability was classified into three types by Hodgkin^3^. Neurons with type III excitability cannot generate repetitive firing even for large depolarization currents within a biophysically relevant range, and they fail to form a well-defined frequency-current curve^5^, which is different from repetitive firing for type I and II excitabilities^3,6^. For type I excitability, the resting state changes to repetitive firing with an arbitrarily low firing frequency^3,5–7^. For type II excitability, the resting state switches to repetitive firing with a certain non-zero frequency^3,5–7^. Depending on their excitabilities, neurons exhibit different firing frequency responses to periodic stimulus, distributions of interspike intervals to noise, properties of stochastic resonance, and precision of spike timing^2,8–10^, which is important for neural information processing. For example, different biophysical basis such as internal and external currents and dynamical behaviors to sustained stimulations of three types of excitability have been identified^6,7^. The transition between types of excitability can be induced by changes of ionic currents^6,11–13^, external synaptic inputs^7^, and autaptic currents^15,16^. There have been many investigations of the physiological significance^6,7,14^ and dynamics^11,15,17–20^ of different excitabilities. For example, a type I neuron is similar to a low-pass filter tuned to lower frequencies, and a type III neuron to a band-pass filter tuned to higher and lower frequencies^2^. From the theory of nonlinear dynamics, type I and II excitability correspond respectively to saddle-node bifurcation on an invariant circle and Hopf bifurcation^5,21–23^. Complex bifurcations^12,24,25^, including high-codimension bifurcations^11,17,19^ related to the transition between type I and type II excitability, have been identified.

Compared with type I and II excitability, there are far fewer investigations of type III excitability^8–10,26,27^. Neurons with type III excitability only fire a spike or a few spikes at the onset of step depolarization current stimulation, which is also known as phasic firing^3,5,10^. Type III excitability has been observed in neurons such as the spinal cord neuron^6^, dorsal root ganglion neuron^28^, and auditory brain stem neuron^29–31^. Neurons with type III excitability exhibit extraordinary temporal precision for phase locking^32^ and enhanced coincidence detection^2,9,27^. These properties are related to some physiological^29,31,33^ and pathological functions^28,34^. For instance, for dorsal root ganglion neurons, cellular changes induce a change from type III to type II excitability, which may result in neuropathic pain and muscle spasm^28^. The nucleus laminaris neurons in the auditory brain stem exhibit type III excitability and spike with high timing precision^32,33^, which contributes to sound localization, whereas repetitive firing will degrade sound localization. For nucleus laminaris neurons, brief and repetitive excitatory stimulations can elicit repetitive firing, but constant depolarization current evokes only a single spike^31^. In addition, the transition between types of excitability can be induced by changes of ionic currents. For example, type III excitability can transition to type II excitability, and to type I excitability by adding low-threshold outward currents such as M-type potassium currents, or blocking low-threshold inward currents such as L-type calcium currents and persistent sodium currents^6^. However, when adding low-threshold outward currents such as M-type potassium currents or blocking low-threshold inward currents such as calcium currents, type I excitability is changed to type II excitability, and to type III excitability^6^. Type II excitability is changed to type I excitability, and back to type II excitability by enhancing the conductance of A-type potassium current and non-inactivating calcium current^13^. From the viewpoint of nonlinear dynamics, type III excitability corresponds to a stable steady state, and no bifurcations appear for any constant depolarization currents in the physiological range^3,5,10^, which is different from type I and II excitability. With application of any constant currents within the physiological range to a steady state, neurons with type III excitability initiate to fire a spike and change to a different steady state corresponding to the constant stimulation current. For that reason, the steady state is stable and no bifurcations occur for type III excitability, and bifurcations have not been identified in the transition between type III excitability and the other two types of excitability^5,10^. A fast excitatory autapse has recently been identified as inducing type III excitability to change to type II excitability^26^, which implies that the fast autapse may be a negative factor for the maintenance of type III excitability, and can largely counterbalance the distinction between type II and III excitability. The results show that type II and III excitability have similar responses to the fast excitatory autapse.

The autapse of a neuron is a special synapse that connects to the neuron itself^35^, and has been identified in many types of neurons in brain regions, including the hippocampus^36^, cerebral cortex^35^, and neocortex^37,38^. The autapse plays important roles in modulating firing activities. For example, an inhibitory autapse can promote spike-timing precision^38^ and suppress repetitive firing^37^, and an excitatory autapse can elicit persistent firing^39^. The effect of excitatory autapses on bursting patterns have been reported in biological experiments^40^. In theoretical models^41–51^, the autapse is identified as influencing electronic activities of single neurons such as an excitability switch^15,16^ or resonance^52,53^, and spatiotemporal behaviors of neuronal networks such as spiral waves^54–56^ and synchronization^57,58^. For example, an inhibitory autapse with time delay, which corresponds to a slow autapse, can enhance firing frequency, which is a novel phenomenon different from the common viewpoint that inhibitory effects should induce the reduction of firing frequency^59^. An inhibitory autapse can induce the enhancement of signal transmission in neuronal networks^60^, and the enhancement of bursting^61^ has been simulated and analyzed in theoretical models. An excitatory or inhibitory autapse can induce a transition between type I and II excitability^15,16^. An excitatory autapse can induce the reduction of the number of spikes within a burst, in contrast to the traditional viewpoint that an excitatory effect should induce the enhancement of firing frequency^62^. Slow- and fast-decay inhibitory autapses can induce a depolarization block (resting state with high potential) to change to firing and subthreshold oscillation^63,64^, respectively, which shows that slow- and fast-decay autapses have different effects on electronic activities. These phenomena are abnormal behaviors induced by excitatory or inhibitory autapses. Except for the autapse, excitatory or inhibitory effects such as external stimulation or coupling are identified to induce abnormal phenomena. For example, inhibitory stimulation can enhance neuronal firing^65,66^, and inhibitory coupling can enhance the firing frequency of a neuronal network^67–69^. Such results of abnormal activities of neural firings enrich the knowledge of neurodynamic and nonlinear dynamics.

Based on the three viewpoints that an excitatory autapse can induce abnormal dynamical behaviors^59,62– 69^, fast-decay autapses may have similar effects to type II and III excitability^26^, and slow-decay autapses may have effects on neuronal ring patterns different from fast ones^63,64^, the influences of slow-decay excitatory autapses on firing dynamics of type II and III excitability are investigated in the present paper and compared with fast-decay excitatory autapses. Four important results are obtained. First, based on a previous study indicating that the dynamical properties of type II and III excitability respond to sustained stimulation^6^, similar and different responses to current stimulations with brief or long durations between type II and III excitability are acquired and compared with Hopf bifurcation or nullclines. Second, the fast-decay excitatory autapse, which plays a role similar to periodic stimuli of brief duration, can induce a resting state to change to repetitive firing for both type II and III excitability. This resembles the result of a recent investigation^26^ and is consistent with the common viewpoint that excitatory effects can promote neuronal firing activities. Such a result shows that there is no obvious difference in the effects of a fast-decay autapse on type II and III excitability. Third, a slow-decay excitatory autapse can induce a resting state for type II excitability changing to repetitive firing, and for type III excitability changing to a resting state following a transient spike instead of repetitive firing, which shows that a slow-decay autapse has different effects on type II and III excitability. Last, the slower the excitatory autapse the stronger the excitatory effect of the autaptic current. However, for type III excitability, a slow-decay excitatory autapse cannot induce the resting state to change to a stable firing pattern, which is different from the common viewpoint that an excitatory effect should enhance neural firing activities. Such a result shows that the dynamical behavior of type III excitability with a slow-decay excitatory autapse is paradoxical, in contrast to the traditional viewpoint. This phenomenon is explained by the responses of the resting state for type III excitability to the autaptic current of a slow-decay autapse.

## Results

### Type II and III excitability in the Morris-Lecar model

**Figure 1:**
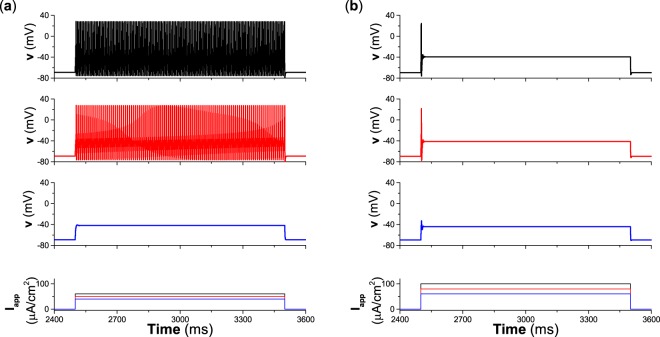
Responses of neurons with different types of excitability to pulse current with long duration 1000 ms and different intensities. (**a**) Type II excitability. (**b**) Type III excitability. The top three panels are membrane potentials induced by the three pulse currents in the bottom panel. The intensities of pulse currents in (**a**) are *I*_*pulse*_ = 60 *μ*A∕cm^2^, *I*_*pulse*_ = 50 *μ*A∕cm^2^, and *I*_*pulse*_ = 40 *μ*A∕cm^2^. The intensities of pulse currents in (**b**) are *I*_*pulse*_ = 100 *μ*A∕cm^2^, *I*_*pulse*_ = 80 *μ*A∕cm^2^, and *I*_*pulse*_ = 60 *μ*A∕cm^2^.

For the Morris-Lecar (ML) neuron with type II excitability, a subthreshold stimulation (*I*_*pulse*_ = 40 *μ*A∕cm^2^ with duration 1000 ms, lower line in the bottom panel of Fig. 1(a)) cannot induce firing, while suprathreshold stimulations (*I*_*pulse*_ = 50 *μ*A∕cm^2^ and 60 *μ*A∕cm^2^ with duration 1000 ms, top two lines in the bottom panel of Fig. 1(a)) can induce repetitive firing, as shown in the second and top panels, respectively, of Fig. 1(a).

For the ML neuron with type III excitability, three stimulation current pulses with different intensities (*I*_*pulse*_ = 60 *μ*A∕cm^2^, 80 *μ*A∕cm^2^, and 100 *μ*A∕cm^2^, duration 1000 ms) are applied, as shown in the bottom panel of Fig. 1(b). When the stimulation is subthreshold (*I*_*pulse*_ = 60 *μ*A∕cm^2^), not a spike but a subthreshold oscillation is induced, as depicted in the third panel of Fig. 1(b). As *I*_*pulse*_ increases to become suprathreshold (80 *μ*A∕cm^2^ and 100 *μ*A∕cm^2^), a spike is induced at the onset of the pulse, as shown in the second and top panels, respectively, of Fig. 1(b). Only one or a few spikes appear at the onset of the depolarization current pulse for type III excitability, and no repetitive spiking appears, which is different from type II excitability.

### Different dynamics between type II and III excitability

**Figure 2:**
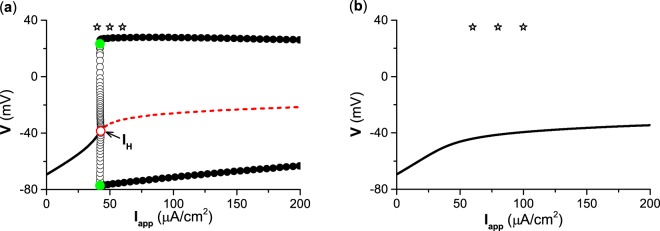
Changes of dynamical behaviors with respect to *I*_*app*_ for the ML model with different types of excitability. (**a**) Bifurcations for type II excitability. The black solid and red dashed curves correspond to the stable and unstable equilibria, respectively. The upper (lower) solid and hollow circles correspond to the maximal (minimal) value of the stable and unstable limit cycles, respectively. The red hollow circles and green solid circles represent the subcritical Hopf bifurcation point *I*_*H*_ = *I*_*app*_ ≈ 42.797 *μ*A∕cm^2^ and saddle-node bifurcation point of the limit cycle *I*_*app*_ ≈ 42.179 *μ*A∕cm^2^, respectively. *I*_*H*_ is the current point at which the Hopf bifurcation occurs. (**b**) Changes of the stable equilibrium for type III excitability. The three stars represent the three *I*_*pulse*_ values used in Fig. 1. The bifurcation diagrams (**a,b**) are obtained using XPPAUT 8.0 (http://www.math.pitt.edu/bard/xpp/xpp.html).

For type II excitability, bifurcations of the membrane potential with increasing depolarization current (*I*_*app*_) are shown in Fig. 2(a). A subcritical Hopf bifurcation occurs at *I*_*H*_ = *I*_*app*_ ≈ 42.797 *μ*A∕cm^2^ (red circle), by which a stable focus (solid black line) changes to an unstable focus (solid red line), and an unstable limit cycle (black hollow cycle) appears. In the present paper, *I*_*H*_ is the current point at which the Hopf bifurcation occurs. The unstable limit cycle and stable limit cycle (black solid cycle) collide and disappear at *I*_*app*_ ≈ 42.179 *μ*A∕cm^2^ (green dot) to form a fold (saddle-node) bifurcation of the limit cycle. The stable limit cycle corresponds to firing. The upper (lower) solid circle corresponds to the maximal (minimal) value of the membrane potential of the stable limit cycle. The red hollow circle and green solid circles correspond to the subcritical Hopf bifurcation point and the fold bifurcation point of the limit cycle. The three stimulation (*I*_*pulse*_) values in Fig. 1(a) are depicted by the three stars in Fig. 2(a). The behavior is a stable focus for the stimulation (*I*_*pulse*_) value corresponding to the first star, and the behaviors are stable firing for the stimulation (*I*_*pulse*_) values corresponding to the second and third stars.

For type III excitability, the resting states for all applied depolarization current (*I*_*app*_) values are stable, and no spiking or bifurcation occurs for any fixed applied depolarization current (*I*_*app*_) values in a physiological range, as shown in Fig. 2(b). The three stimulation (*I*_*pulse*_) values used in Fig. 1(b) are depicted by the three stars in Fig. 2(b). At each stimulation (*I*_*pulse*_) value, the behavior is stable equilibrium.

**Figure 3:**
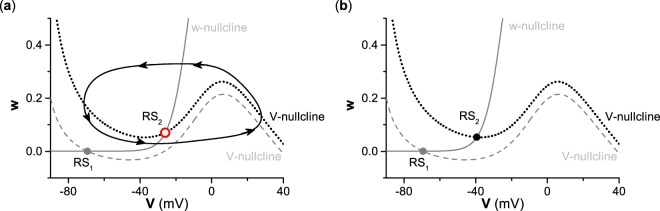
Dynamical behaviors in the phase plane for different types of excitability. (**a**) Type II excitability. The nullclines, equilibria (the stable focus RS_1_ (gray solid dot) for *I*_*app*_ = 0 *μ*A∕cm^2^, and unstable focus RS_2_ (red hollow circle) for *I*_*app*_ = 100 *μ*A∕cm^2^), and limit cycle (black cycle with counterclockwise arrows). (**b**) Type III excitability. The nullclines and equilibria (the stable focus RS_1_ (gray solid dot) for *I*_*app*_ = 0 *μ*A∕cm^2^, and the stable focus RS_2_ (black solid dot) for *I*_*app*_ = 100 *μ*A∕cm^2^). The nullclines $\dot{V}=0$ for *I*_*app*_ = 0 *μ*A∕cm^2^(gray dashed line) and for *I*_*app*_ = 100 *μ*A∕cm^2^ (black dotted line), and the nullcline $\dot{w}=0$ (gray solid curve) for both *I*_*app*_ = 0 *μ*A∕cm^2^ and *I*_*app*_ = 100 *μ*A∕cm^2^.

For neurons with type II excitability, due to the Hopf bifurcation occurring at the applied depolarization current *I*_*app*_ = *I*_*H*_ ≈ 42.797 *μ*A∕cm^2^ (Fig. 2(a)), the point RS_1_ (gray solid dot) is the stable focus for the applied depolarization current *I*_*app*_ = 0 *μ*A∕cm^2^, and RS_2_ (red hollow dot) is the unstable focus for the applied depolarization current *I*_*app*_ = 100 *μ*A∕cm^2^, as shown in Fig. 3(a). The black cycle with counterclockwise arrows represents the stable limit cycle corresponding to spiking for the applied depolarization current *I*_*app*_ = 100 *μ*A∕cm^2^. The nullcline $\dot{w}=0$ for *I*_*app*_ = 0 *μ*A∕cm^2^ is the same as for the applied depolarization current *I*_*app*_ = 100 *μ*A∕cm^2^, as shown by the gray solid curve, since $\dot{w}=0$ is independent from the applied depolarization current *I*_*app*_. The gray dashed curve and dotted curve represent the nullclines of $\dot{V}=0$ for *I*_*app*_ = 0 *μ*A∕cm^2^ and *I*_*app*_ = 100 *μ*A∕cm^2^, respectively, which shows that the position of the nullcline $\dot{V}=0$ moves up as the applied depolarization current *I*_*app*_ increases. The results show that the resting state changes to the stable limit cycle for type II excitability as the applied depolarization current *I*_*app*_ switches from 0 *μ*A∕cm^2^ to 100 *μ*A∕cm^2^.

For type III excitability, the points RS_1_ (gray solid dot) and RS_2_ (black solid dot) are stable equilibria for the applied depolarization current *I*_*app*_ = 0 *μ*A∕cm^2^ and the applied depolarization current *I*_*app*_ = 100 *μ*A∕cm^2^, respectively, as shown in Fig. 3(b), which correspond to the resting states. The point RS_1_ is the intersection point between nullclines $\dot{V}=0$ (gray dashed curve) and $\dot{w}=0$ (gray solid curve) for the applied depolarization current *I*_*app*_ = 0 *μ*A∕cm^2^. When *I*_*app*_ = 100 *μ*A∕cm^2^, the nullcline $\dot{V}=0$ shifts up, as shown by the black dotted curve in Fig. 3(b), and the nullcline $\dot{w}=0$ remains unchanged. The point RS_2_ is the intersection of nullclines $\dot{V}=0$ (black dotted curve) and $\dot{w}=0$ (gray solid curve) for the applied depolarization current *I*_*app*_ = 100 *μ*A∕cm^2^. The equilibria RS_1_ for the applied depolarization current *I*_*app*_ = 0 *μ*A∕cm^2^ and RS_2_ for the applied depolarization current *I*_*app*_ = 100 *μ*A∕cm^2^ are stable because no bifurcations occur with respect to *I*_*app*_ for type III excitability. The results show that the steady-state membrane potential increases for type III excitability as the applied depolarization current *I*_*app*_ switches from 0 *μ*A∕cm^2^ to 100 *μ*A∕cm^2^.

### Different or similar responses to pulse currents for type II and III excitability

**Figure 4:**
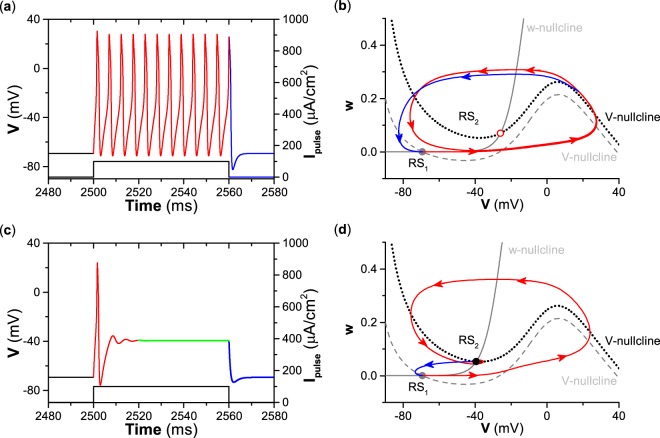
Different responses of the resting state to a pulse current of long duration (60 ms) and corresponding dynamical behaviors in the phase plane for different types of excitability. (**a**) Response for type II excitability. (**b**) Dynamical behaviors in phase plane corresponding to (**a**). (**c**) Response for type III excitability. (**d**) Dynamical behaviors in phase plane corresponding to (**c**).

For type II excitability, repetitive firing can be induced by suprathreshold pulse current stimulation (*I*_*pulse*_ > *I*_*H*_) of long duration. For example, the repetitive firing and dynamical behaviors in the (*V*, *w*)-plane are shown in Fig. 4(a,b), respectively, for a suprathreshold stimulation *I*_*pulse*_ = 100 *μ*A∕cm^2^ of duration 60 ms. Before the pulse stimulation, the membrane potential stays at the resting state corresponding to the stable equilibrium RS_1_ for *I*_*app*_ = 0 *μ*A∕cm^2^. After the application of pulse current, the dynamical behavior corresponding to *I*_*app*_ = 100 *μ*A∕cm^2^ with duration 60 ms begins from RS_1_, evolves to the stable limit cycle (red circle with counterclockwise arrows), and stays at the limit cycle until 60 ms have elapsed, as shown by the red lines in Fig. 4(a,b). The duration 60 ms is much longer than the period of the limit cycle (about 5.32 ms), which results in repetitive spiking (about 12 spikes) appearing within the 60 ms pulse. The interspike interval of repetitive firing is the period of the limit cycle. When the pulse current of duration 60 ms is terminated, the trajectory recovers to the stable equilibrium RS_1_ for *I*_*app*_ = 0 *μ*A∕cm^2^, as shown by the blue curves in Fig. 4(a,b). Such a dynamical mechanism is the cause of the repetitive firing shown in Figs. 1(a) and 4(a). If the pulse current stimulation is not terminated, then the repetitive firing will continue.

For type III excitability, only a single spike can be induced by a suprathreshold stimulation with long duration, e.g., the depolarization pulse current with strength *I*_*pulse*_ = 100 *μ*A∕cm^2^ and duration 60 ms shown in Fig. 4(c,d). The evolution process of the membrane potential has three phases, which are labeled by red, green, and blue, as indicated below. The trajectory begins from the stable equilibrium RS_1_ for *I*_*app*_ = 0 *μ*A∕cm^2^, runs across the middle branch and to the right branch of the nullcline $\dot{V}=0$ (gray dashed curve) to form a spike, and evolves to the stable equilibrium RS_2_ for *I*_*app*_ = 100 *μ*A∕cm^2^, which is the first phase (red curve) which takes about 20 ms. In the second phase (green curve), the trajectory stays at RS_2_ because the equilibrium RS_2_ is stable, as shown in Fig. 4(c). The suprathreshold stimulation means that the stimulation can induce the trajectory to run across the middle branch of the nullcline $\dot{V}=0$. When the stimulation is terminated, the trajectory evolves from the equilibrium RS_2_, recovers to the equilibrium RS_1_, and stays at RS_1_, which is the last phase (blue curve), as depicted in Fig. 4(c,d). The result shows that the single spike is a transient behavior induced by the suprathreshold stimulation, which is the beginning phase of the dynamical behavior as *I*_*app*_ switches from 0 *μ*A∕cm^2^ to 100 *μ*A∕cm^2^.

**Figure 5:**
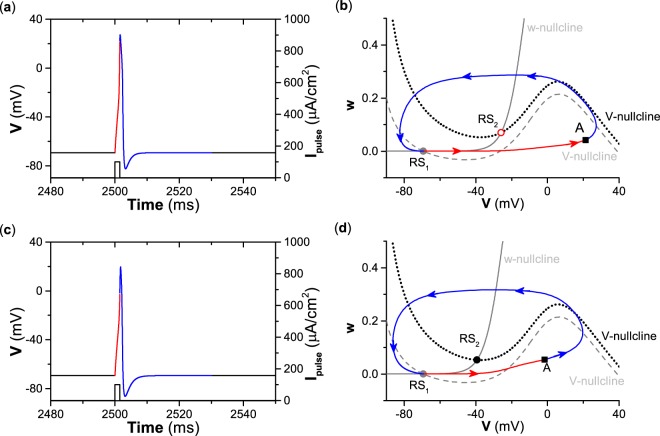
Similar responses of the resting state to a pulse current of brief duration (1.5 ms) and the corresponding dynamical behaviors in the phase plane for different types of excitability. (**a**) Response for type II excitability. (**b**) Dynamical behaviors in phase plane corresponding to (**a**). (**c**) Response for type III excitability. (**d**) Dynamical behaviors in phase plane corresponding to (**c**).

The responses of the resting state to a suprathreshold pulse current with brief duration are similar for both type II and III excitability, as shown in Fig. 5. For example, with stimulation *I*_*pulse*_ = 100 *μ*A∕cm^2^ of a brief duration of 1.5 ms, a single spike is elicited for both type II and III excitability, as shown in Fig. 5(a,c), with corresponding dynamical behaviors in the (*V*, *w*)-plane, as shown in Fig. 5(b,d). The trajectory begins from the stable equilibrium (RS_1_) for *I*_*app*_ = 100 *μ*A∕cm^2^, runs across the middle branch of the nullcline $\dot{V}=0$ (gray dashed curve), and evolves to point *A* (square) when the pulse stimulation is terminated, as shown by the red curve in Fig. 5(b,d). Point *A* is located to the right of the middle branch of the nullcline $\dot{V}=0$; therefore, the trajectory after point *A* can run across the right branch of nullcline $\dot{V}=0$ for both *I*_*app*_ = 0 *μ*A∕cm^2^ and *I*_*app*_ = 100 *μ*A∕cm^2^ to form a spike, and then it evolves to and remains at the stable equilibrium RS_1_, as depicted by the blue curves in Fig. 5(b,d). Different from a pulse current of long duration, the trajectory induced by a pulse current of brief duration cannot stay at the stable limit cycle for type II excitability or at the stable equilibrium RS_2_ for type III excitability because the pulse duration of 1.5 ms is too short.

**Figure 6:**
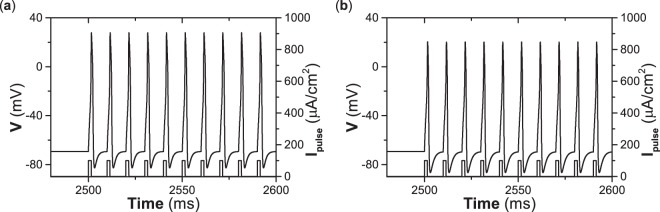
Responses of resting state to periodic pulse currents of brief duration. (**a**) Type II excitability. (**b**) Type II excitability. Pulse duration 1.5 ms and period 11.5 ms.

From Fig. 5(a,c), it can be speculated that periodic pulse currents with suprathreshold strength can induce repetitive spiking for both type II and III excitability. For example, when periodic current pulses with *I*_*pulse*_ = 100 *μ*A∕cm^2^, brief duration 1.5 ms, and period 11.5 ms are applied to the resting state, a repetitive spiking pattern is induced, and each pulse can induce a spike for both type II and III excitability, as shown in Fig. 6(a,b), respectively. Comparing Fig. 6(a,b), the firing patterns induced by the periodic pulse currents are similar for both types of excitability.

The interspike interval of the repetitive firing induced by the periodic pulse currents is nearly equal to the period of the pulse currents, which is different from the repetitive firing induced by the suprathreshold current of long duration, where the interspike interval is nearly equal to the period of the limit cycle for type II excitability.

Comparing Figs. 4, 5, and 6, it can be concluded that periodic pulse currents of brief duration can induce repetitive firing for both type II and III excitability, and the interspike interval of the repetitive firing nearly equals the period of the stimulation. For type II excitability, a current higher than *I*_*H*_ ≈ 42.797 *μ*A∕cm^2^ can induce repetitive firing whose interval equals the period of the limit cycle bifurcated from the Hopf bifurcation. However, for type III excitability, a suprathreshold current of long duration cannot induce repetitive firing, and it induces just a single spike. Such results can be used to recognize the autapse-induced dynamical behaviors of the ML model with type II or III excitability. For convenience, the repetitive firing induced by periodic pulse currents of brief duration is called case-1 firing, and that induced by current higher than *I*_*H*_ ≈ 42.797 *μ*A∕cm^2^ (stimulation with long duration) is called case-2 firing, and this appears for only type II excitability. The results also show that responses of the resting state to current stimulation of long duration are different for type II and III excitability, while current stimulations of brief duration are similar for both.

Another issue should be emphasized. For a neuron with type II excitability, a pulse current of long duration (Fig. 4(a)) provides more excitatory stimulations than one of brief duration (Fig. 5(a)). Correspondingly, a pulse current of long duration can elicit more spikes than one of brief duration. However, type III excitability has a different result. Although the pulse current of long duration shown in Fig. 4(c) provides more excitatory stimulations than the one of brief duration depicted in Fig. 5(c), the former cannot induce more spikes than the latter.

### Autapse induced-dynamical behaviors

**Figure 7:**
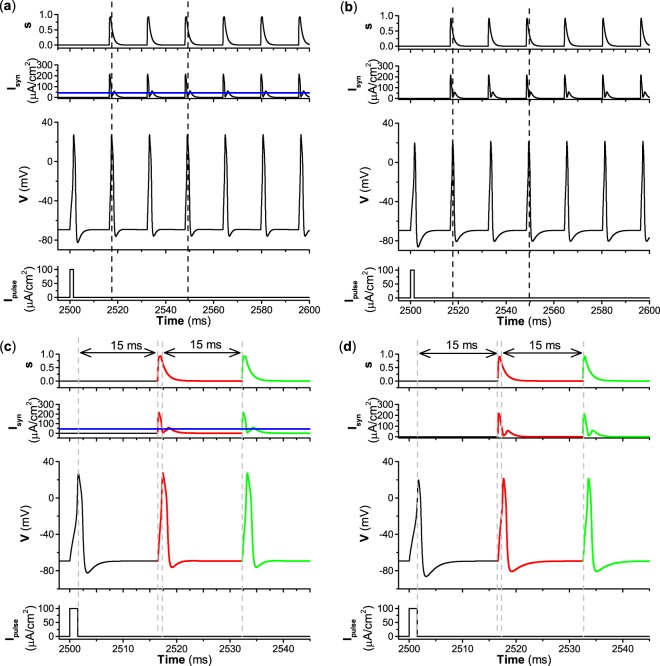
Excitatory autapse with fast decay rate (*β* = 1) induces similar repetitive firings (case-1) for different types of excitability (**a**) Type II excitability. (**b**) Type III excitability. (**c**) Enlargement of (**a**); (**d**) Enlargement of (**b**). Other parameters: *g*_*syn*_ = 3.0 mS∕cm^2^ and *τ* =  7 ms. The blue curves in (**a,c**) correspond to the value of Hopf bifurcation *I*_*H*_ ≈ 42.797 *μ*A∕cm^2^.

**Figure 8:**
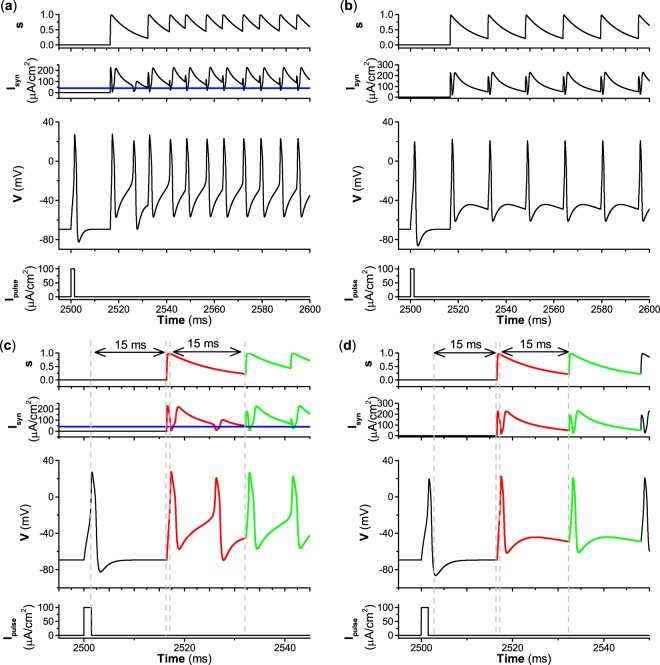
Excitatory autapse with relatively slow decay rate (*β* = 0.1) induces different repetitive firings for type II and III excitability. (**a**) Type II excitability and case-2 firing. (**b**) Type III excitability and case-1 firing. (**c**) Enlargement of (**a**); (**d**) Enlargement of (**b**). Other parameters: *g*_*syn*_ = 3.0 mS∕cm^2^ and *τ* = 15 ms. The blue curves in (**a,c**) correspond to the value of Hopf bifurcation *I*_*H*_ ≈ 42.797 *μ*A∕cm^2^.

**Figure 9:**
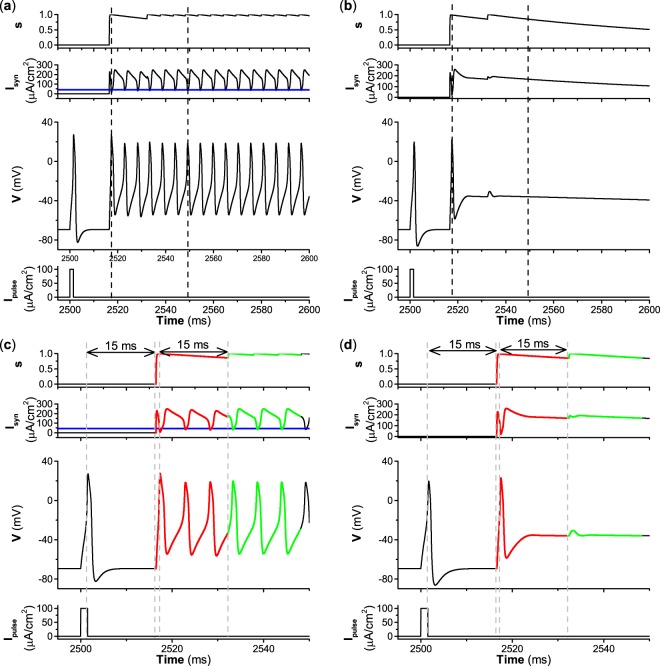
Excitatory autapse with slow decay rate (*β* = 0.01) induces different dynamical behaviors for type II and III excitability. (**a**) Repetitive firing (case-2 firing) for type II excitability. (**b**) No stable repetitive firing for type III excitability. (**c**) Enlargement of (**a**). (**d**) Enlargement of (**b**). Other parameters: *g*_*syn*_ = 3.0 mS∕cm^2^ and *τ* = 15 ms. The blue curves in (**a,c**) correspond to the value of Hopf bifurcation *I*_*H*_ ≈ 42.797 *μ*A∕cm^2^.

By varying the decay rate *β*, excitatory autapses can induce different behaviors for both type II and III excitability with time delay *τ* = 15 ms and autaptic conductance *g*_*syn*_ = 3.0 mS∕cm^2^. The behaviors of the ML neuron induced by an excitatory autapse when *β* = 1, *β* = 0.1, and *β* = 0.01 are shown in Figs. 7, 8, and 9, respectively. The first spike is elicited by a pulse current with strength *I*_*pulse*_ = 100 *μ*A∕cm^2^ and a brief duration of 1.5 ms, as shown in the bottom panels of Figs. 7–9. For the relatively large decay rate *β* = 1, the repetitive firing induced by the excitatory autapse is similar for type II and III excitability, as shown in the third panels of Fig. 7(a,b). The first spike can activate the autaptic current (*I*_*syn*_ = − *g*_*syn*_*s*(*t* − *τ*)(*V* − *E*_*syn*_)), which is delayed *τ* = 15 ms and can evoke the second spike. The autaptic current induced by the second spike increases quickly and decreases relatively quickly to form a pulse-like current, which is mainly determined by the fast decay (*β* = 1) of the variable *s*, as shown in the top panels of Fig. 7(a,b). The pulse-like autaptic current induced by the second spike can induce the third spike. So, the autaptic current induced by the *k*-th spike can induce the (*k* + 1)-th spike (*k* =1, 2, 3, …), which leads to repetitive firing. In the process, each pulse-like autaptic current can induce a spike, which resembles the periodic pulse currents with brief duration depicted in Fig. 6. Therefore, an important characteristic of repetitive firing is that the interspike interval of the firing nearly equals both the period of the autaptic current and the time delay *τ*.

As the decay rate *β* decreases to 0.1, i.e., the excitatory autapse becomes relatively slow, the excitatory autapse can induce repetitive firing for type II and III excitability, as shown in Fig. 8(a–d). Fig. 8(c,d) are enlargements of Fig. 8(a,b), respectively. Although the autapse induces repetitive firing for both excitabilities, the dynamical mechanism of repetitive firing differs for type II and III excitability. Due to the relatively slow decay rate (*β* = 0.1) of the variable *s*, the autaptic current (red curve) decays relatively slowly. For type II excitability, the first spike can activate the autaptic current, which is delayed 15 ms and can induce the second spike. Then the autaptic current (red curve in Fig. 8(c)) can induce the third spike because the autaptic current *I*_*syn*_ decays slowly and is larger than *I*_*H*_ ≈ 42.797 *μ*A∕cm^2^ within the duration of the time delay *τ* = 15 ms. After the third spike, the autaptic current within an oscillation period shorter than the time delay is higher than *I*_*H*_ in most time durations, and can induce spikes to produce repetitive firing. *I*_*syn*_ does not resemble the periodic pulse-like currents with period nearly equal to the time delay *τ*, and is higher than *I*_*H*_ ≈ 42.797 *μ*A∕cm^2^ (blue line in Fig. 8(a,c)) in most time durations. Therefore, the repetitive firing for type II excitability is induced by current larger than *I*_*H*_, and resembles case-2 firing, as shown in Fig. 4(a). An important characteristic of the repetitive firing is that the interspike interval (7.72 ms) does not equal the time delay (15 ms), and is a certain value determined by both the period of the limit cycle and the value of *I*_*syn*_. However, for type III excitability, the autaptic current *I*_*syn*_ exhibits periodic pulse-like currents with period nearly equal to the time delay, and the repetitive firing resembles the case-1 firing shown in Fig. 6. The interspike interval (15.72 ms) of case-1 firing nearly equals the time delay (15 ms). Therefore, for a relatively slow autapse, the repetitive firing for type II excitability resembles case-2 firing, while that for type III excitability is similar to case-1 firing.

As the decay rate *β* decreases to 0.01, i.e., the excitatory autapse becomes slow, the autapse-induced dynamical behavior for type II excitability differs from that for type III excitability. The autapse induces repetitive firing for type II excitability and cannot induce repetitive firing for type III excitability, as shown in Fig. 9(a–d). Fig. 9(c,d) are enlargements of Fig. 9(a,b), respectively. The repetitive firing for type II excitability resembles case-2 firing because the autaptic current *I*_*syn*_ is larger than *I*_*H*_ (blue lines in the second panels of Fig. 9(a,c)) in most time durations. Correspondingly, the interspike interval (5.27 ms) of the repetitive firing is a certain value that does not equal the time delay (15 ms) and is determined by both the period of the limit cycle and the value of the autaptic current *I*_*syn*_. The dynamical mechanism of the repetitive firing is the same as for the repetitive firing, as shown in Fig. 8(a). However, for type III excitability, the autaptic current decreases very slowly due to the slow decay of the variable *s*, and remains large before the next activation of autaptic current. When the second spike activates the autapse, the variable *s* increases to a small extent, which results in small increases of the autaptic current, which cannot induce a spike. After the second spike, the autaptic current decays very slowly. Therefore, the autaptic current cannot induce the third spike. This result shows that an autapse with a slow decay rate fails to elicit repetitive firing for type III excitability.

### Effect of decay rate *β* on dynamical behaviors in the plane (*τ*, *g*_*syn*_)

**Figure 10:**
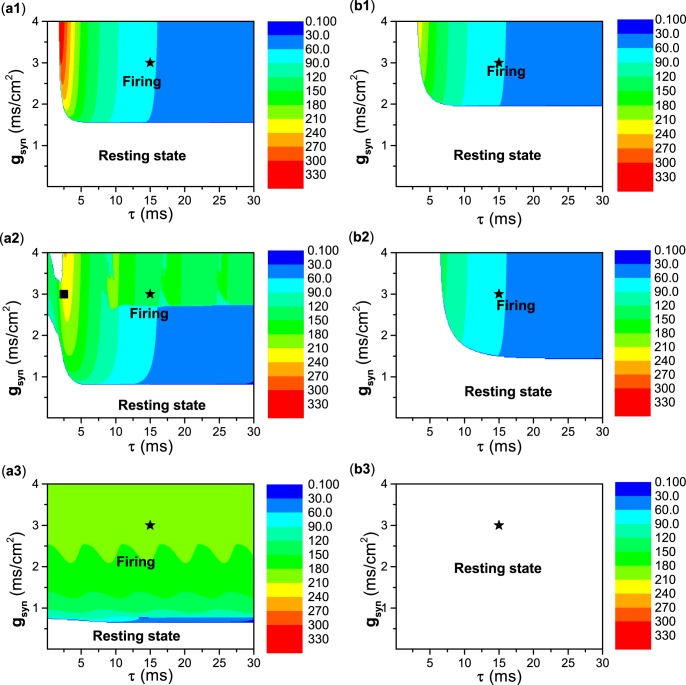
Distributions of resting state (blank region) and firing (color region) in the plane (*τ*, *g*_*syn*_) at different *β* values. Type II excitability (left) and type III excitability (right). (a1) and (b1) *β* = 1. (a2) and (b2) *β* = 0.1. (a3) and (b3) *β* = 0.01. Color scales represent the frequency of neuronal firing. Stars in (a1) and (a2) correspond to parameter values of left and right panels of Fig. 7, stars in (b1) and (b2) correspond to parameter values of left and right panels of Fig. 8, and stars in (c1) and (c2) correspond to parameter values of left and right panels of Fig. 9, square in (a2) corresponds to parameter values of Fig. 11.

As the decay rate *β* changes, the distributions of the resting state and firing on the plane (*τ*, *g*_*syn*_) for type II and III excitability are as shown in Fig. 10.

For a relatively large decay rate, e.g., *β* = 1, the distributions of the resting and firing states for type II excitability resemble those for type III excitability, as shown in the top panels of Fig. 10. The firing is induced by an autaptic current with periodic pulse-like characteristics, as shown in Fig. 7, which resembles case-1 firing (Fig. 6). The firing shown in Fig. 7 corresponds to the stars shown in Fig. 10(a1,a2).

**Figure 11:**
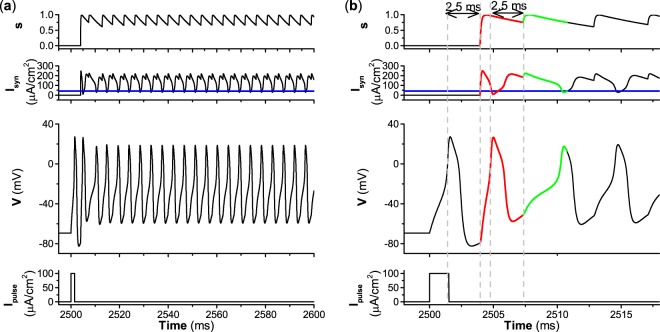
Case-2 firing induced by excitatory autapse with relatively slow decay rate (*β* = 0.1) and small time delay (*τ* = 2.5 ms) for type II excitability. (**a**) *g*_*syn*_ = 3.0 mS∕cm^2^; (**b**) Enlargement of (**a**). Blue curves correspond to the value of Hopf bifurcation *I*_*H*_ ≈ 42.797 *μ*A∕cm^2^.

As the decay rate *β* decreases to a relatively small value, e.g., *β* = 0.1, the parameter region of firing enlarges for type II excitability and narrows for type III excitability, as shown in the middle panels of Fig. 10. The result for type III excitability is simple. The firing patterns locate at the upper-right in Fig. 10(b2), and are induced by an autaptic current with a periodic pulse-like current, which resembles case-1 firing. The case-1 firing shown in Fig. 8(b,d) corresponds to the star shown in Fig. 10(b2). For type II excitability, the results are complex. The firing locates at the top of Fig. 10(a2) and has two cases. (1) For the autaptic conductance *g*_*syn*_ < 2.7 mS∕cm^2^ and time delay *τ* > 5 ms, the autaptic current exhibits a periodic pulse-like characteristic, and the interspike interval of the firing pattern nearly equals the time delay *τ*. The firings resemble case-1 firing. (2) For the remaining parameter region of firings, the autaptic current is greater than *I*_*H*_ ≈ 42.797 *μ*A∕cm^2^, and the firings resemble case-2 firing. When *g*_*syn*_ > 2.7mS∕cm^2^ and *τ* > 5 ms, the firings resemble those shown in Fig. 8(a,c), with parameters corresponding to the star shown in Fig. 10(a2). For time delay *τ* < 5 ms, a representative example of case-2 firing is shown in Fig. 11, and the parameters correspond to the square shown in Fig. 10(a2).

Comparing Fig. 10(a2,a1), it can be found that the parameter region of firing increases as the decay rate *β* decreases for type II excitability. The increase is seen in both the time delay *τ* and autaptic conductance *g*_*syn*_. However, comparing Fig. 10(b2,b1), it can be found that the parameter region of firing enlarges as *β* increases for type III excitability, and is induced by the increase of the time delay threshold for firing.

As the decay rate further decreases, e.g., *β* = 0.01, the dynamical behavior for type II excitability is different from that for type III excitability in the parameter region shown in Fig. 10(a3,b3) (*τ* < 30 ms). For type II excitability, the firing behavior covers most regions of the plane, as shown in Fig. 10(a3). However, the firing behavior disappears in the plane for type III excitability, as depicted in Fig. 10(b3). The disappearance of firing behavior for type III excitability corresponds to the dynamical behavior shown in Fig. 9(b,d). For type II excitability, the case-1 firing appears in a narrow parameter region in blue in Fig. 10(a3). The case-2 firing corresponds to the green region in Fig. 10(a3).

Comparing Fig. 10(a3,a2), it can be found that the parameter region of firing behavior enlarges with decreasing *β* for type II excitability, which is induced by the decrease of *g*_*syn*_ for firing. However, comparing Fig. 10(b1–b3), the disappearance of firing behavior is induced by the increase of the time delay threshold for firing, hence no firing appears when *τ* < 30ms.

### Paradoxical phenomenon for type III excitability

Comparing the autaptic current between the black dashed lines in the second panels of Figs. 7(b) and 9(b), we find that although the autaptic current *I*_*syn*_ for the decay rate *β* = 0.01 (Fig. 9(b)) is much stronger than for *β* = 1 (Fig. 7(b)), no action potentials can be induced for *β* = 0.01 because only a large, rapid change of the autaptic current *I*_*syn*_ can induce an action potential for type III excitability. Thus, for type III excitability, a stronger excitatory effect (*β* = 0.01) of the autaptic current *I*_*syn*_ cannot induce repetitive firing, while a weaker excitatory effect can do so, which differs from the traditional viewpoint that a strong excitatory effect should enhance firing activities.

**Figure 12:**
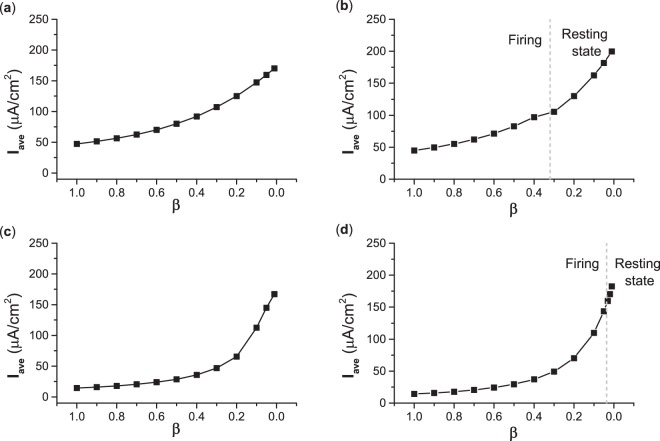
Effect of *β* on average autaptic current within two interspike intervals to represent the excitatory effect when *g*_*syn*_ = 3.0 mS∕cm^2^. (**a**) Type II excitability when *τ* = 4 ms. (**b**) Type III excitability when *τ* = 4 ms. (**c**) Type II excitability when *τ* = 15 ms. (**d**) Type III excitability when *τ* = 15 ms.

To quantitatively measure the excitatory effect of *I*_*syn*_, the average autaptic current within two black dashed lines in Figs. 7 and 9 (between two interspike intervals for the firing induced by the fast-decay autapse) is calculated. For type II excitability, the average autaptic current within the two black dashed lines increases from 14.2 for *β* = 1 (Fig. 7(a)) to 167.29 for *β* = 0.01 (Fig. 9(a)). For type III excitability, the average autaptic current within the two black dashed lines increases from 14.18 for *β* = 1 (Fig. 7(b)) to 182.36 for *β* = 0.01 (Fig. 9(b)). The changes of the average autaptic current with respect to the decay rate *β* for type II and III excitability are shown in Fig. 12(a,b) (the autaptic conductance *g*_*syn*_ = 3.0 mS∕cm^2^, and the time delay *τ* = 4 ms) and Fig. 12(c,d) (*g*_*syn*_ = 3.0mS∕cm^2^, *τ* = 15 ms), respectively. With decreasing *β*, the autapse becomes slower and the average autaptic current increases, which means a stronger excitatory effect of autaptic current *I*_*syn*_ for both type II and III excitability. However, the excitatory effect of the autaptic current induces different responses between type II and III excitability. For example, for type II excitability, the excitatory effect of the autaptic current can induce firing when the decay rate *β* ∈ (0.01, 1) for *g*_*syn*_ = 3.0 mS∕cm^2^ and *τ* = 4 ms. Such a result for type II excitability is consistent with the common viewpoint that the excitatory effect usually facilitates neuronal firing activities. However, for type III excitability, the excitatory effect of autaptic *I*_*syn*_ can induce firing only when *β* ∈ (0.32, 1), and cannot induce firing for *β* ∈ (0.01, 0.32) when *g*_*syn*_ = 3.0 mS∕cm^2^ and *τ* = 4 ms. For type III excitability, a stronger excitatory effect cannot induce firing, while a weaker excitatory effect can. The border between firing and non-firing is depicted by the vertical dashed line in Fig. 12(b,d). Such a result for type III excitability contrasts with the common viewpoint of the excitatory effect, which presents a novel characteristic of type III excitability and nonlinear phenomena.

## Discussion and Conclusion

The autapse is a self-feedback connection of neurons^35^, and it plays important roles in modulation of neuronal electronic activities^26,38,39,41,51^, which are complex due to the nonlinearity of neurons and autapses. Based on bifurcation or nullcline, similar or different responses of the resting state to current stimulations of brief or long duration between type II and III excitability are identified. Comparing dynamical characteristics of autaptic currents with those of current stimulations, the effects of fast- and slow-decay excitatory autapses on the firing activities of the ML model with type II and III excitability are identified in the present paper.

For both type II and III excitability, a fast-decay excitatory autapse can induce changes from the resting state to repetitive firing, similar to the findings of a previous report^26^. These results are consistent with the common viewpoint that the excitatory effect should enhance neural firing activities. In biological experimental studies, nucleus laminaris neurons exhibit type III excitability, and can generate repetitive firing under brief and repetitive excitatory stimulations^31^. For a fast-decay excitatory autapse, the autaptic current exhibits a pulse-like characteristic, which induces similar responses for both type II and III excitability. Furthermore, the slow-decay excitatory autapse has different effects on type II and III excitability^26^ because type II excitability corresponds to Hopf bifurcation, while no bifurcations correspond to type III excitability. The current value corresponding to Hopf bifurcation points for type II excitability is *I*_*H*_. For an excitatory autapse with a slow decay characteristic, the autaptic current *I*_*syn*_ is larger than *I*_*H*_ in most time durations, which leads to repetitive firing. For type III excitability, a slowly changing autaptic current induces a transient spike instead of repetitive firing. Specifically, the autaptic variable *s* activated by a spike remains at a high level before being reactivated by another spike, hence the variable *s* changes little. The small change of autaptic current influenced by the variable *s* cannot induce action potentials, which is the dynamical mechanism of the resting state following a transient spike induced by a slow-decay autapse for type III excitability. The result of the present paper is largely consistent with the result of different responses of type II and III excitability to sustained stimulation reported in a previous study^6^. For a fast decaying autapse, the autaptic currents are similar to periodic stimuli of short duration. However, a slowly decaying autapse behaves as sustained stimulation. Thus, understanding the dynamics of the responses to periodic and sustained stimulus suffices to understand the responses of two types of excitability to autapses with different decaying kinetics, at least for the simplistic model utilized in the present study. The dynamical mechanisms that explain the different responses to slow-decaying autaptic stimulation of type II and III excitability are similar to that explain the different responses to sustained stimulation: destabilization of a fixed point through a subcritical Hopf bifurcation for type II excitability and a fixed point remaining stable (a quasi separatrix crossing mechanism of spike initiation) for type III excitability^6^. The results of the present paper also show that slow- and fast-decay autapses have different effects on neural firing or oscillations, which is consistent with previous studies^63,64^. One property of type III excitability is coincident detection. That is, type III neurons respond only to temporally coincident stimulations (with large amplitudes). Our results indicate that a slow-decay autapse can maintain the property of type III excitability, which is consistent with experimental studies showing that excitatory autapses can enhance coincident detection in a neocortical pyramidal neuron^40^.

The slow-decay excitatory autapse-induced resting state following a transient spike for type III excitability is a paradoxical phenomenon. The smaller the decay rate the slower the autaptic current decays. That is, a slow-decay excitatory autapse provides a stronger excitatory effect than a fast one. However, a fast-decay autapse can induce repetitive firing, while a slow-decay autapse instead induces a resting state following a transient spike. Such a result is different from the traditional viewpoint that strong excitation should induce enhancement of firing activities. In a recent report on abnormal phenomena induced by an excitatory autapse, the excitatory effect was identified to reduce the number of spikes within a burst^62^. Therefore, a novel example in contrast to the common viewpoint of the excitatory effect is given in the present paper. The novel cases of abnormal phenomena induced by excitatory or inhibitory effects should be investigated.

Type III excitability has been found to be involved in some functions^30,31,33^ and diseases^34,70^ of the nervous system. The cellular excitability changes from phasic firing corresponding to type III excitability to repetitive firing corresponding to type II excitability, which is related to neuropathic pain in dorsal root ganglion neurons^34,70^. The excitatory autapse with slow decay characteristic is helpful for the maintenance of type III excitability, which may alleviate muscle spasm and neuropathic pain. In fact, for neurons with type II or III excitability and without autapses, the equations to describe the autapse can be taken as an effective feedback modulation measure, which can be achieved in biological experiments with a dynamic patch clamp or in a circuit implementing the nervous system. The results of the present paper reveal that different effects of fast- and slow-decay excitatory autapses or feedback to the resting state of type II and III excitability are important to adjust neural dynamical behaviors, or may be useful for neuronal information processing.

In the present paper, a single-compartmental phenomenological model neuron with different types of excitability, which receives a non-plastic excitatory synaptic input via autaptic connection or feedback current, is used to simulate biological phenomena, and is simple enough to recognize complex dynamical behaviors. However, to further identify how biophysically different neurons operate under temporally complex, natural synaptic excitation is important to recognize biophysically complex and diverse biological neurons. The behavior of the theoretical model (such as the multiple-compartmental model) under more complex, aperiodic stimuli and/or incorporating analysis of the effects of synaptic plasticity should be investigated.

## Material and Methods

### The ML model

The modified ML model^6^ is described as

1 $$C\dot{V}={I}_{app}-{g}_{Na}{m}_{\infty }(V)(V-{E}_{Na})-{g}_{k}w(V-{E}_{K})-{g}_{L}(V-{E}_{L}),$$

2 $$\dot{w}={\phi }_{w}\frac{{w}_{\infty }(V)-w}{{\tau }_{w}(V)},$$

where ${m}_{\infty }(V)=0.5[1+\tanh (\frac{V-{\beta }_{m}}{{\gamma }_{m}})]$; ${w}_{\infty }(V)=0.5[1+\tanh (\frac{V-{\beta }_{w}}{{\gamma }_{w}})]$; ${\tau }_{\infty }(V)={\cosh }^{-1}(\frac{V-{\beta }_{w}}{{\gamma }_{w}})$; *V* is membrane potential; *w* is the activation of the delayed rectifier potassium channel; $\dot{V}$ and $\dot{w}$ are the time-derivatives of *V* and *w*, respectively; *g*_*Na*_ and *E*_*Na*_ are the maximum conductance and reversal potential, respectively, of the sodium current; *g*_*k*_ and *E*_*K*_ are respectively the maximum conductance and reversal potential of the delayed rectifier potassium current; *g*_*L*_ and *E*_*L*_ are respectively the maximum conductance and reversal potential of the leakage current; *C* is the membrane capacitance; and *I*_*app*_ is the applied depolarization current.

In the present paper, two kinds of applied current are chosen. *I*_*app*_ is constant and is a bifurcation parameter, and *I*_*app*_ is pulse current stimulation with strength *I*_*pulse*_ and duration Δ*t* to induce the changes of dynamical behavior of the ML model. For example, if a pulse with *I*_*pulse*_ = 100 *μ*A∕cm^2^ and duration Δ*t* = 60 ms is applied to the ML model, then the dynamical behavior corresponding to *I*_*app*_ = 0 *μ*A∕cm^2^ is changed to that corresponding to *I*_*app*_ = 100 *μ*A∕cm^2^ for 60 ms, and then it returns to behavior corresponding to *I*_*app*_ = 0 *μ*A∕cm^2^.

### ML model with excitatory autapse

When the current *I*_*syn*_ mediated by an autapse is introduced to Eq. 1 while Eq. 2 remains unchanged, the ML model with autapse is formed, and is described as

3 $$C\dot{V}={I}_{app}-{g}_{Na}{m}_{\infty }(V)(V-{E}_{Na})-{g}_{k}w(V-{E}_{K})-{g}_{L}(V-{E}_{L})+{I}_{syn},$$

4 $$\dot{w}={\phi }_{w}\frac{{w}_{\infty }(V)-w}{{\tau }_{w}(V)}.$$

The autaptic current *I*_*syn*_ is described by

5 $${I}_{syn}=-{g}_{syn}s(t-\tau )({V}_{pos}-{E}_{syn}),$$

where *g*_*syn*_ is the autaptic conductance, *E*_*syn*_ is the reversal potential of the autapse, *V*_*pos*_ is the postsynaptic membrane potential, *τ* is the time delay due to the time lapse occurring in synaptic processing, and *s* is the activation variable of the synapse, determined as

6 $$\dot{s}=\alpha \Gamma ({V}_{pre})(1-s)-\beta s,$$

where *V*_*pre*_ is the presynaptic membrane potential; Γ(*V*_*pre*_) is the sigmoid function of *V*_*pre*_; and *α* and *β* are the rise and decay rates, respectively, of synaptic activation. In the nervous system, the deactivation time of the NMDA receptors of an excitatory autapse ranges from  ~ 10 to  ~ 100 ms, and can be modulated by the values of *β*. The smaller the *β* value the slower the decay of the autapse. Γ(*V*_*pre*_ − *θ*_*syn*_) = 1∕(1 + *exp*(−10(*V*_*pre*_ − *θ*_*syn*_))), where *θ*_*syn*_ is the synaptic threshold, which is set at a suitable value to ensure that the synaptic transmitter release occurs only when the presynaptic neuron generates a spike, i.e., when *V*_*pre*_ > *θ*_*syn*_. In Eqs. 5 and 6, *V*_*pre*_ = *V*_*pos*_ = *V* to ensure the synapse is an autapse.

The parameter values of the autapse are set as follows: *E*_*syn*_ = 30 mV to ensure that the autapse is excitatory, *θ*_*syn*_ = 10 mV, and *α* = 12. The parameters *g*_*syn*_, *τ*, and *β* are chosen as control parameters to modulate the effects of the excitatory autapse.

### Parameters for type II and III excitability

The ML neuron model can exhibit firing properties with different excitabilities by modulating its intrinsic parameters, including *β*_*w*_^6^ and *β*_*m*_^18^. In the present study, the values of *β*_*w*_ are selected to ensure that the model exhibits type II and III excitability. *β*_*w*_ = − 25 mV for type III excitability and *β*_*w*_ = − 13 mV for type II excitability. Other parameters for both type III and II excitability are set as: *g*_*Na*_ = 20 ms∕cm^2^, *g*_*K*_ = 20 ms∕cm^2^, *g*_*L*_ = 2 ms∕cm^2^, *E*_*Na*_ = 50 mV, *E*_*K*_ = − 100 mV, *E*_*L*_ = − 70 mV, *C* = 2 *μ*F∕cm^2^, *β*_*m*_ = − 1.2 mV, *γ*_*m*_ = 18 mV, *γ*_*w*_ = 10 mV, and *ϕ*_*w*_ = 0.15. *I*_*app*_ is chosen as the control parameter to modulate the dynamical behavior of the model.

### Methods

The equations are integrated using the fourth-order Runge-Kutta method with integration step 0.01 ms, and bifurcation diagrams are obtained using XPPAUT 8.0^71^, which is freely available at http://www.math.pitt.edu/bard/xpp/xpp.html.
